# Surveillance of impact of PCV-10 vaccine on pneumococcal meningitis in Mozambique, 2013 – 2015

**DOI:** 10.1371/journal.pone.0177746

**Published:** 2017-06-12

**Authors:** Aquino Albino Nhantumbo, Goitom Weldegebriel, Reggis Katsande, Linda de Gouveia, Charlotte Elizabeth Comé, Arlindo Zacarias Cuco, Vlademir Vicente Cantarelli, Cícero Dias, Juliana Caierão, Jason Mwenda Mathiu, Eduardo Samo Gudo

**Affiliations:** 1 Laboratório Nacional de Referência de Microbiologia, Instituto Nacional de Saúde, Ministério da Saúde, Maputo, Mozambique; 2 World Health Organization African Regional Office, Harare, Zimbabwe; 3 National Institute for Communicable Diseases (NICD) of the National Health Laboratory Service (NHLS), Johannesburg, South Africa; 4 Universidade Feevale, Rio Grande de Sul, Brazil; 5 Universidade Federal de Ciências de Saúde de Porto Algre (UFCSPA), Porto Alegre, Brazil; 6 Instituto Nacional de Saúde, Ministério da Saúde, Maputo, Mozambique; Universidade de Lisboa Faculdade de Medicina, PORTUGAL

## Abstract

**Background:**

Vaccination using the 10-valent conjugate vaccine (PCV-10) was introduced into the Extended Program on Immunization in Mozambique in March 2013, however its impact on pediatric pneumococcal meningitis is unknown. In this study, we assessed for the first time the impact of PCV10 on the burden of pneumococcal meningitis in children less than 5 years of age at the three largest hospitals in Mozambique.

**Method:**

Between March 2013 and December 2015, a total of 744 cerebrospinal fluid (CSF) samples were collected from eligible children, of which 160 (21.5%) were positive for *S*. *pneumoniae*. Of these, only 86 samples met the criteria for serotyping and were subsequently serotyped using sequential multiplex PCR (SM-PCR), but 17 samples were non-typable.

**Results:**

The proportion of cases of pneumococcal meningitis decreased from 33.6% (124 of 369) in 2013 to 1.9% (3 of 160) in 2015 (*p* < 0.001). The relative frequency of PCV10 serotype cases also decreased from 84.2% (48 of 57) in 2013 to 0% (0 of 3) in 2015 (*p* = 0.006). Between 2013 and 2015, serotype coverage of PCV-10 and PCV13 vaccine formulations was 66.7% and 81.2%, respectively.

**Conclusion:**

Altogether, our findings shows that introduction of PCV-10 immunization resulted in rapid decline of pneumococcal meningitis children less than 5 years old in Mozambique. This decline was accompanied by substantial changes in the pattern of circulating pneumococcal serotypes.

## Background

Pneumococcal disease remains a main public health concern worldwide, despite the availability of effective vaccine. The disease is associated with high rates of long-term disability and case-fatality rate [1–4]. The pneumococcal conjugate vaccines (PCVs) has been shown to be safe and highly effective in preventing invasive pneumococcal disease (IPD) and reducing the burden of pneumococcal disease in children, both in developed and developing countries [1–6]. In sub-Saharan Africa, which carries the highest burden of the disease worldwide, many countries have recently introduced PCVs into their childhood vaccination programs [7].

Of remark, previous studies have shown that despite the fact that introduction of PCV led to a decline in the burden of pneumococcal meningitis caused by those serotypes that are covered by the PCV [1,2,4,5,7], on the other hand, it resulted in relative increase in the burden of pneumococcal meningitis caused by non–PCV serotypes. Thus, serotype-specific pneumococcal surveillance is key to assess and understand the impact of PCV in the epidemiology of pneumococcal meningitis, including to assess the potential replacement of PCV serotypes by non-PCV serotype, following the introduction of PCV [1–12].

Different vaccine formulations that vary in their composition in term of serotype coverage are available [10–12]. In Mozambique, 10-valent pneumococcal conjugate vaccine (PCV10) was introduced into the Extended Program on Immunization (EPI) in 2013 [13]. This vaccine formulation covers the 10 most common pneumococcal serotypes globally (serotypes 4, 6B, 9V, 14, 18C, 19F, 23F, 1, 5, 7F). However, the 13-valent PCV (PCV13) vaccine formulation is now available, which provides added coverage for the following serotypes 3, 6A and 19A to PCV10 [9,11,12]. A recent publication by our group showed that PCV10 formulation in use in Mozambique cover 81.8% of circulating serotypes in the country [14]. However, the impact of PCV10 immunization in Mozambique on the burden of pneumococcal disease and on the serotype distribution since the introduction of this vaccine is not yet known. But data from other countries demonstrated that this vaccine lead to rapid decline on *S*. *pneumoiae* related meningitis among children with acute bacterial meningitis (ABM) [11,12]. In this regard, we conducted the present study to assess the impact of PCV10 on the burden and serotype distribution of *S*. *pneumoniae* among children with suspected ABM.

## Methods

### Study design, study sites and target population

A cross-sectional study was conducted between March 2013 and December 2015 using data from the routine sentinel surveillance for paediatric ABM. Sentinel surveillance system for ABM in Mozambique comprises three sentinel sites which are regional hospitals, namely, Maputo Central Hospital (HCM), Beira Central Hospital (HCB) and Nampula Central Hospital (HCN), situated in the southern, central, and northern regions of the country, respectively [13]. The sample for this study consisted of all hospitalized children (<5 years old) with laboratory confirmed pneumococcal meningitis by culture and/or PCR. The study was divided into 2 periods: baseline period, which corresponded to the year in which PCV10 was introduced in Mozambique (2013), and the follow up period, which corresponded to the period post introduction of PCV10 (2014–2015). We used serotype-specific data from March 2013 to March 2014 as previously reported by *Nhantumbo et al* [14] as the baseline for the comparison with the period post introduction of PCV10, in order to assess the impact of PCV10 on the burden and serotype distribution of pneumococcus.

### Ethics approval and consent to participate

The study was approved by the Mozambican National Bioethics Committee (Ref #: IRB00002657). Verbal consent was obtained from the legal representative of each child as per the requirements of the routine sentinel surveillance system for acute bacterial meningitis in Mozambique. Only verbal consent was obtained to avoid significant interference in the routine care being provided to these patients at these sites. A logbook was used to record all patients who consented to participate.

### Case definition

Case definition in use in Mozambique for sentinel surveillance of ABM follows World Health Organization (WHO) guidelines as previously described [13]. A confirmed case of pneumococcal meningitis (PM) was defined as the presence of *S*. *pneumoniae* identified either by culture or multiplex qPCR in cerebrospinal fluid (CSF) [13,14].

### Samples collection and questionnaire

Basic demographic data (age, gender, PCV vaccination status) and clinical presentation were recorded using a standard case investigation form. Samples were collected and sent to the clinical microbiology laboratory at each sentinel sites for microbiological culture as previously described by *Nhantumbo et al* [13].

### Laboratory testing

#### *S*. *pneumoniae* identification by culture and PCR

Pneumococcal isolates were first identified at the clinical laboratories at each sentinel sites, using standard culture methods as previously described [14]. All pneumococcal isolates and negative CSF samples were sent to a single reference laboratory in the country (National Reference Microbiology Laboratory—NMRL) for confirmation using culture and multiplex qPCR assay for simultaneous detection of the following target genes: *lytA* for *S*.*pneumoniae*, *hpd* for *H*.*influenzae* and *ctrA* for *N*.*meningitidis*. For multiplex qPCR assay, genomic DNA was extracted from all pneumococcal isolates and/or CSF samples. PCR reactions conditions were performed as previously described [14]. For detection of pneumococcus using PCR, all samples with cycle threshold (ct) value ≤ 35 were considered positive.

#### Serotyping of *S*. *pneumoniae*

All pneumococcal isolates and *S*. *pneumoniae* positive CSF samples were shipped to the National Institute for Communicable Diseases (NICD) of the National Health Laboratory Service (NHLS) in South Africa and also to Universidade Federal de Ciências de Saúde de Porto Alegre (UFCSPA), Brazil for serotyping.

CSF samples and isolates from 2013 were shipped to Brazil and tested at UFCSPA for determination of the serotype of pneumococci using the sequential multiplex PCR (SM-PCR) as described by *Nhantumbo et al* [14] and Carvalho *et al* [15]. These data were used as a baseline in our study because PCV was introduced into the EPI in 2013. On the other hand, samples from 2014 and 2015 were shipped to South Africa and tested at NICD for determination of serotype of pneumococci using a real-time PCR assay consisting of 7 triplexed reactions to identify 40 serotypes/serogroups representing the majority of disease-causing isolates of *Streptococcus pneumoniae*. This assay targets the 13 serotypes included within the 13-valent conjugate vaccine and 8 additional key serotypes or serogroups. The capsular polysaccharide (*cps*) was used as internal control [16]. Since the method used in Brazil (UFCSPA) was based on conventional PCR, a cutoff of ≤ 30 was used (our own unpublished observation) to select samples for serotyping. On the other hand, in South Africa (NICD), the cutoff of ≤ 35 was used to identify eligible samples for serotyping, because the method in use was based on real-time PCR, which is known to be highly sensitive. The reference strain *S*. *pneumoniae* ATCC 49619 was used as the quality control.

### Statistical analysis

Data were entered into a database developed using Epi Info version 3.5.4 (CDC, U.S.A.) and was analysed using SPSS statistical software version 20 (IBM, U.S.A.). Categorical variables were reported as proportion and statistical significance differences were assessed using Fisher’s exact test with a significance level of less than 0.05.

## Results

### General characteristics of study participants

**Fig 1:**
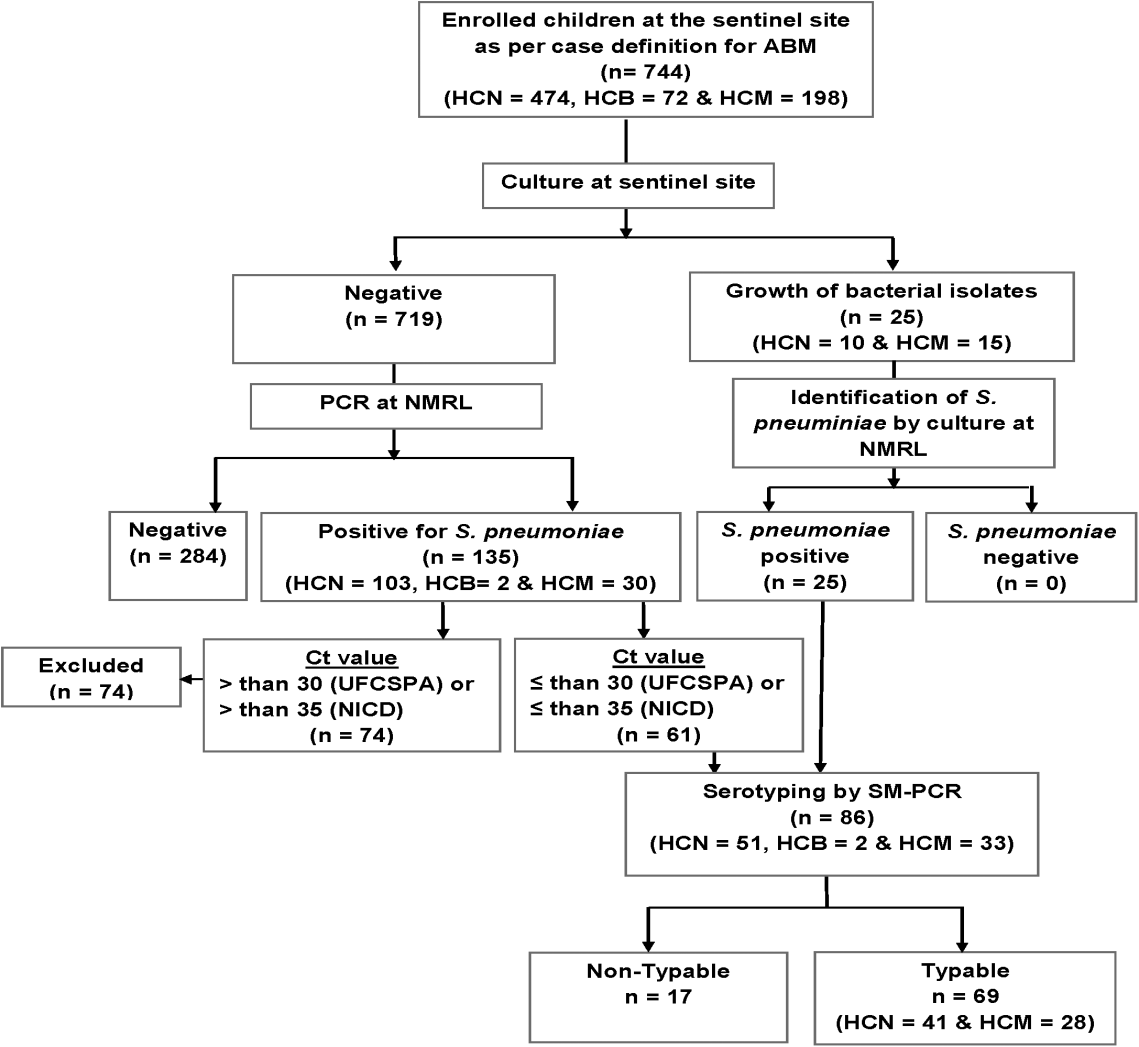
Flowchart of sample collection and testing. The flow chart depicts the number of CSF samples collected and tested between March 2013 and December 2015. CSF: Cerebrospinal fluid; HCM: Hospital Central de Maputo; HCB: Hospital Central da Beira; HCN: Hospital Central de Nampula; SM-PCR: sequential multiplex polymerase chain reaction; NMRL: National Reference Microbiology Laboratory.

Between March 2013 and December 2015, a total of 744 CSF samples were collected from children clinically suspected of ABM, who were admitted at the sentinel sites. The median age of study participants was 12 months (IQR 5–36 months) and 53.8% (400/744) of them were male. More than half of the children (61.3%, 456/744) were aged less than 24 months, while children of the age group 24–59 months comprised 38.7% (288/744) of the study group (see Table 1). Most of participants were from Nampula Central Hospital (63.7%, 474/744), while children from Maputo Central Hospital and Beira Central Hospital represented 26.6% (n = 198) and 9.7% (n = 72), respectively (Fig 1).

**Table 1:** Age and gender distribution of enrolled patients between 2013 and 2015.

Characteristics		Total	2013		2014		2015	
			ABM	*S*.*p*. positive	ABM	*S*.*p*. positive	ABM	*S*.*p*. positive
**Total**		744	369	124 (33.6%)	215	33 (15/3%)	160	3 (1.9%)
**Age in month, median (IQR)**		12 (5–36)	9 (4–35)	7.50 (5–19.50)	20 (6–43)	11 (5–30)	21 (7.50–36)	36 (2–36)
**Age categories**								
	0–11 months	348 (46.8%)	208 (56.4%)	81 (65.3%)	88 (40.9%)	17 (51.5%)	52 (32.5%)	1 (33.3%)
	12–23 months	108 (14.5%)	43 (11.6%)	19 (15.3%)	25 (11.6%)	5 (15.2%)	40 (25.0%)	0 (0.0%)
	24–59 months	288 (38.7%)	118 (32.0%)	24 (19.4%)	102 (47.4%)	11 (33.3%)	68 (42.5%)	2 (66.7%)
**Gender**								
	Male	400 (53.8%)	193 (52.3%)	68 (54.8%)	125 (58.1%)	19 (57.6%)	82 (51.2%)	2 (66.7%)
	Female	344 (46.2%)	176 (47.7%)	56 (45.2%)	90 (41.9%)	14 (42.4%)	78 (48.8%)	1 (33.3%)

### Prevalence of *S*. *pneumoniae* among children with ABM declined from 2013 to 2015

*S*. *pneumoniae* was identified in a total of 160 (21.5%) samples, of which 25 were initially identified by culture and the remaining 135 were identified by PCR (see Fig 1). *H*. *influenzae* and *N*. *meningitides* were identified in a total of 6.3% (47 of 744) and 2.6% (19 of 744) of children with ABM, respectively (S1 Dataset).

**Fig 2:**
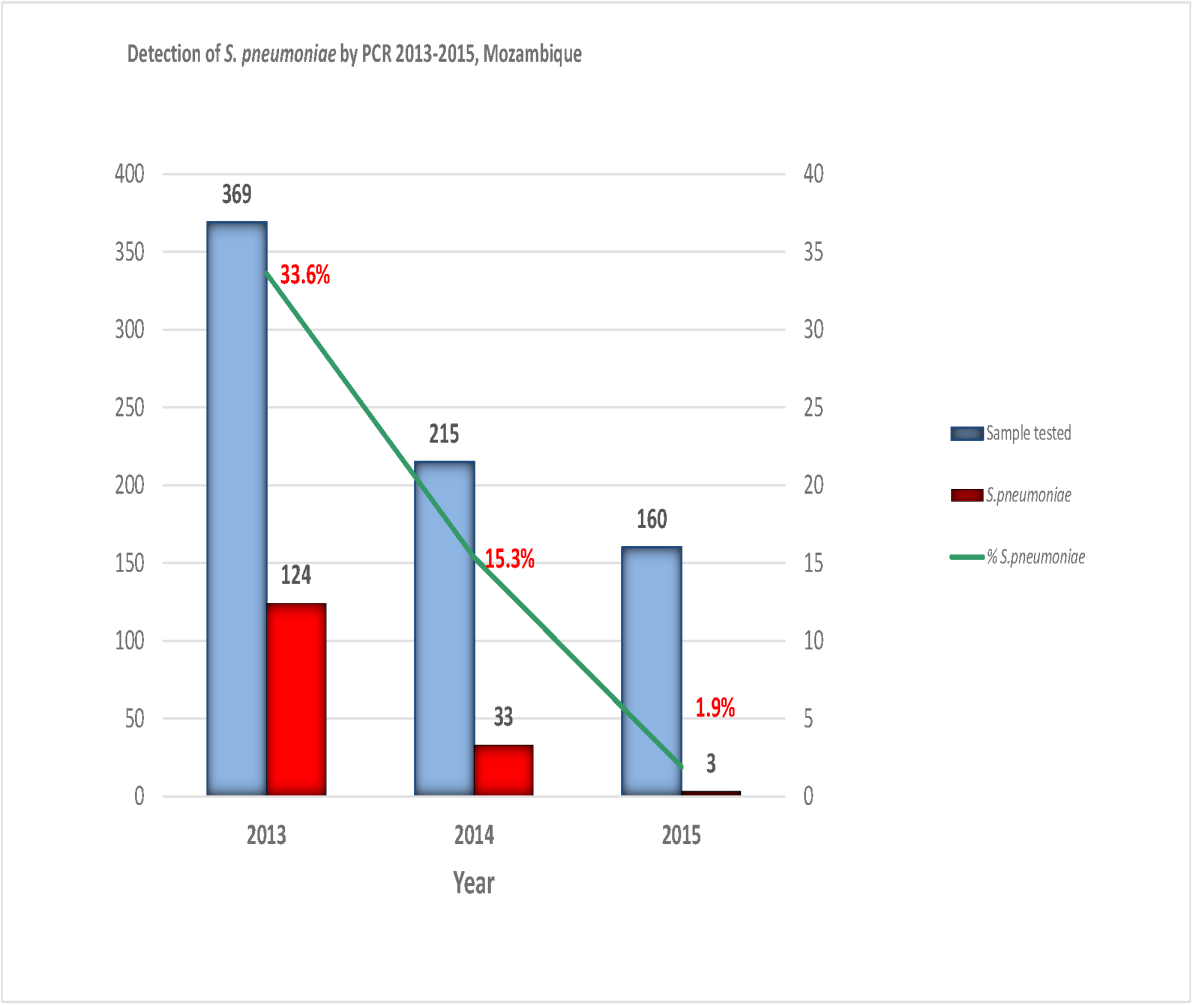
Detection of *S*. *pneumoniae* by PCR from 2013–2015, Mozambique. Figure depicts the annual variation of the relative frequency of *S*. *pneumoniae* causing pneumococcal meningitis and also the variation in the number of CSF samples collected from children <5 years. Frequency of *S*. *pneumoniae* was determined using polymerase chain reaction (PCR). CSF: cerebrospinal fluid.

Between 2013 and 2015 there was a decline in the number of reported ABM cases from 369 in 2013 to 160 in 2015. Decline in reported cases of ABM was accompanied by decline in the relative frequency of pneumococcal meningitis cases, which decreased significantly from 33.6% in 2013 to 1.9% in 2015 (*p* < 0.001) (Fig 2).

### Serotype distribution, coverage rate of pneumococcal vaccine formulations and impact of PCVs on pneumococcal meningitis

**Fig 3:**
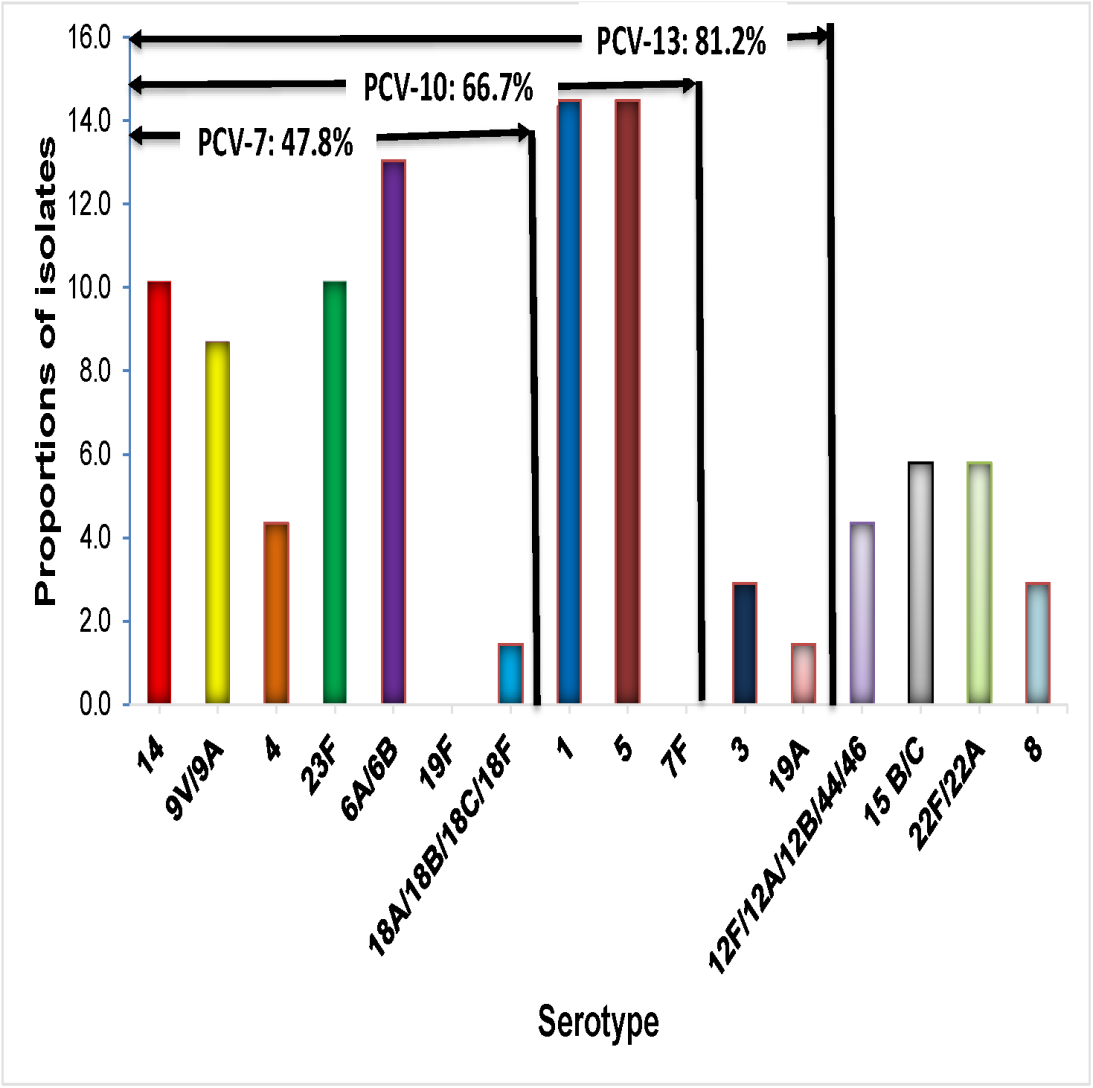
Distribution of serotypes of *S*. *pneumoniae* and vaccine coverage rates for PCV-7, PCV-10 and PCV-13 vaccine formulations. Each bar represents the relative frequency of each serotype of *S*. *pneumoniae*. The value in the arrows above the bars depicts the vaccine coverage rates for PCV-7, PCV-10 and PCV-13, respectively NV, serotypes not included in 13-valent pneumococcal conjugate vaccine. NV, nonvaccine serotypes (serotypes 12F/12A/12B/44/46, 8, 22F/22A and 15B/C).

Serotyping of *S*. *pneumoniae* was performed in a total of 86 samples, comprising the 17 isolates of *S*. *pneumoniae* identified by culture and 69 PCR positive CSF samples, for which their ct value was below the cut off (ct ≤ 30 for samples tested at UFCSPA and ct ≤ 35 for samples tested at NICD). Of the 86 samples for which serotyping was performed, a total of 69 (80.2%) were typable and their corresponding serotype was identified, but the remaining 17 samples were not-typeable (SM-PCR NT), as shown in Fig 1. Overall, the most frequent serotypes were 1 (14.5%), 5 (14.5%), 6A/6B (13.0%), 23F (10.1%), 14 (10.1%), 9V/9A (8.7%), 4 (4.3%), 3 (2.9%), 19A (1.4%), 1.4 (18A/18B/18C/18F) and remaining 18.8% (13/69) were non-PCV serotypes (including serotypes 8, 22F/22A, 15 B/C and 12F/12A/12B/44/46) (Fig 3). Table 2 shows that the largest share of PCV10 serotype cases decreased from 84.2% (48 of 57) in 2013 to 0% (0 of 3) in 2015 (*p* = 0.006).

**Table 2:** Annual serotypes distribution of *Streptococcus pneumoniae* from pneumococcal meningitis in Mozambique, 2013–2015.

PCR results (NICD/Brazil)	Year			Total
Serotypes	2013 (n = 57)	2014 (n = 9)	2015 (n = 3)	
**PCV7**				
14	7	0	0	7
9V/9A	5	1	0	6
4	3	0	0	3
23F	7	0	0	7
6A/6B	9	0	0	9
19F	0	0	0	0
18A/18B/18C/18F	1	0	0	1
**PCV10**				
1	7	3	0	10
5	9	1	0	10
7F	0	0	0	0
**PCV13**				
3	2	0	0	2
19A	1	0	0	1
Non PCV serotypes*	6	4	3	13

* Non PCV serotypes includes: serotypes 8, 12F/12A/12B/44/46, 15 B/C and 22F/22A

Fig 3 shows that the rate of vaccine coverage against the serotypes of *S*. *pneumoniae* causing paediatric meningitis in Mozambique was 47.8% (33/69), 66.7% (53/69), and 81.2% (56/69) for PCV-7, PCV-10, and PCV-13, respectively.

The non-PCV serotypes during 2013–2015 were 8, 22F/22A, 15 B/C and 12F/12A/12B/44/46 (Table 2).

**Fig 4:**
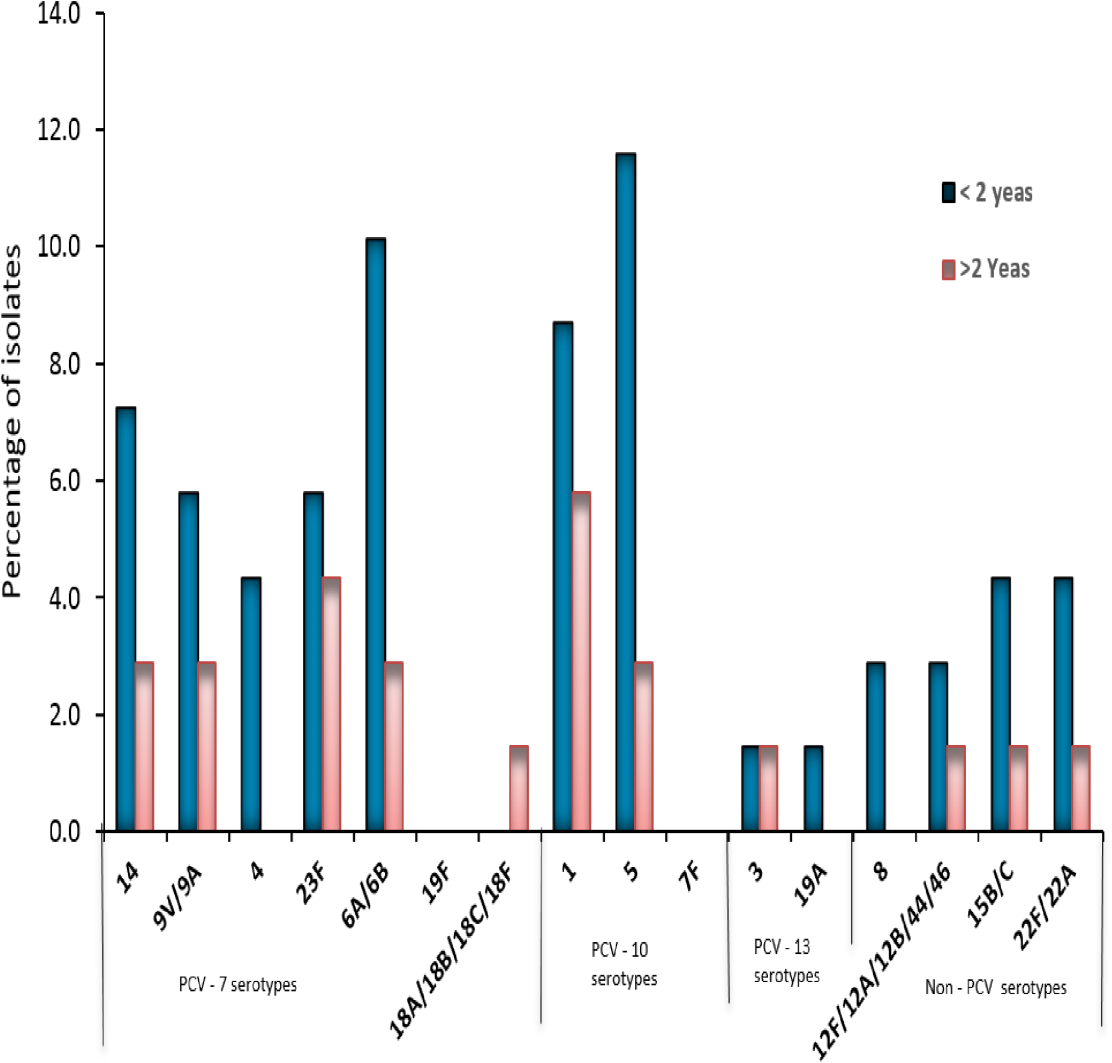
Proportions of isolates causing pediatric pneumococcal meningitis stratified by pneumococcal serotype and age. Each bar represents the relative frequency of each serotype of *S*. *pneumoniae*.

We analysed the serotype distribution of pneumococcus strains stratified by age and found that in children younger than 2 years old, the proportions of almost all PCV serotypes was higher than that found in children older than 2 years old (Fig 4).

## Discussion

In Mozambique, the PCV10 vaccine formulation was introduced into the Expanded Program of Immunization (EPI) in 2013, but no data is available on the impact of this vaccine on the burden of pneumococcal meningitis. In this study, we assessed for the first time the trend of pneumococcal meningitis as well as the change in the distribution of serotypes of *S*. *pneumoniae* in the period between 2013 and 2015 to determine the impact of PCV immunization in the country. Data from our study showed a remarkable reduction in the relative frequency of pneumococcal meningitis during the first three years after introduction of PCV10 in the EPI program in Mozambique. Routine data on vaccine coverage obtained from EPI program in Mozambique demonstrated that during this period, the coverage of PCV10 was 97%, which gives more consistency to findings from our study. Finding from this study is similar to that reported in studies conducted in developed countries such as USA [17], UK [18], Denmark [19], England and Wales [20] and Finland [21], as well as in developing countries such as South Africa [22] and Kenya [23]. In these countries, there was a rapid decline of pneumococcal meningitis including all other invasive forms of pneumococcal disease few years after introduction of pneumococcal vaccine.

We observed a longitudinal change in the serotype distribution of *S*. *pneumoniae* after the introduction of the PCV10. Although, serotype 1 and 5 remained the most common serotype from 2013 to 2014, therefore they declined significantly from 2014 to 2015 (Table 2). These findings are in line with previous observations, and underline the efficacy of vaccination strategies to strengthen herd protection [21–22].

In regard to serotype coverage of PCV10 and PCV13, data from our study shows that this vaccines formulation covers 66.7% (53/69) and 81.2% of circulating serotypes, respectively. These rates are lower than those reported in a recently published paper from Mozambique, which reported coverage rates of PCV10 and PCV13 of 81.8% and 93.9%, respectively [14]. The decline in the serotype coverage of PCVs may be related to serotype replacement phenomenon. Similar findings were found in a previous study conducted in South Africa [24], Denmark [19], England and Wales [20].

In fact, we observed an emergence of non-vaccine serotypes (NV), such as serotypes 12, 15B/C, 8, 22F/22A, 12F/12A/12B/44/46, which occurred in 18.8% of the children with confirmed pneumococcal meningitis. In addition, the increasing trend in the proportion of non-PCV10 serotypes, suggests that the replacement of serotypes is ongoing, although the absolute rates remained low. Non-vaccine serotypes has increased following the introduction of PCVs in several countries of sub-Saharan Africa [24,25], Denmark [19], England and Wales [20].

Data from our study suggest that PCV10-related serotypes declined since introduction of PCV10. Similar results were found in previous studies conducted in Brazil [26], South Africa [24] and Finland [27], which is an indicative of the effectiveness of this vaccine in reducing the burden of these serotypes.

We would like to acknowledge some limitations of our study, which may influence the interpretation of the results. For instance, our data were collected at three largest hospitals in Mozambique, which may pose problems of representativeness. Secondly, the poor quality of information recorded in the case investigation forms limited our ability to assess the outcomes and vaccination status of the children. Third, our molecular serotyping assay targets only 40 of the most common serotypes/serogroups, and was not able to serotype few samples, most probably because the corresponding serotypes were not included in our multiplex PCR. Lastly, although the methods used in Brazil (conventional PCR) and South Africa (real-time PCR) were different, the decline in the frequency of serotypes between 2013 and subsequent years (2014 and 2015) cannot be attributed to the use of different PCR methods, as conventional PCR (used during the baseline in 2013) is in general known to be less sensitive than real time PCR (used in 2014 and 2015, respectively).

## Conclusion

Our study provides preliminary evidence on the impact of pneumococcal vaccination on pneumococcal meningitis in Mozambique, showing a rapid and consistent decline in the frequency of pneumococcal meningitis, accompanied by more discrete change in the serotype distribution and serotype coverage over the first three years post introduction of PCV10 formulation into the EPI in Mozambique.

## Consent for publication

Our manuscript does not present any individual person's data, however, consent to participate was obtained from each participant as state in the ethics statement section.

## Supporting information

S1 Dataset Database of the Pediatric Bacterial Meningitis (PBM), 2013–2015. (XLSX)

## Abbreviations

ABM: acute bacterial meningitis
ATCC: american type culture collection
CLSI: laboratory standards institute guidelines
CSF: cerebrospinal fluid
ct: cycle threshold
DNA: deoxyribonucleic acid
dNTP: deoxynucleoside triphosphate
HCN: nampula central hospital
INS: National Institute of Health
IPD: invasive pneumococcal diseases
IQR: interquartile range
LP: lumbar puncture
MDRSP: multidrug-resistant strains
MIC: minimum inhibitory concentrations
M-qPCR: multiplex qualitative polymerase chain reaction
NRML: national reference microbiology laboratory
PCV-10: ten-valent conjugate vaccine
PCV-13: thirteen -valent conjugate vaccine
SM-PCR NT: not-typeable by sequential multiplex polymerase chain reaction
SM-PCR: sequential multiplex polymerase chain reaction
S.p: Streptococcus pneumoniae
UDG: uracil DNA glycosylase
UFCSPA: Universidade Federal de Ciências de Saúde de Porto Alegre
